# Fabrication and characterization of small-caliber nanofibrous vascular scaffolds with sustained release of endothelial cell derivatives and heparin

**DOI:** 10.3389/fbioe.2026.1746603

**Published:** 2026-02-26

**Authors:** Ying Wang, Yawen Wang, Qihan Yuan, Jiaoyan Qiu, Jing Wang, Yuanfei Wang, Manfei Fu, Tong Wu

**Affiliations:** 1 Medical Research Center, The Affiliated Hospital of Qingdao University, Qingdao University, Qingdao, China; 2 Shandong Key Laboratory of Medical and Health Textile Materials, Collaborative Innovation Center for Eco-textiles of Shandong Province and the Ministry of Education, College of Textile & Clothing, Qingdao University, Qingdao, China; 3 School of Rehabilitation Sciences and Engineering, University of Health and Rehabilitation Sciences, Qingdao, China; 4 Qingdao Traditional Chinese Medicine Hospital, Qingdao Hiser Hospital Affiliated of Qingdao University, Qingdao, China

**Keywords:** coaxial-emulsion electrospinning, core-shell structure, ECd, heparin, small-caliber vascular scaffolds

## Abstract

**Introduction:**

Cardiovascular diseases are a leading cause of mortality, and artificial blood vessels as an alternative strategy are extensively used in clinical settings. Due to the underlying potential for thrombus formation and intimal hyperplasia, the clinical applications of small-caliber (<6 mm) artificial vessels are limited. Promoting rapid endothelialization and enhancing anticoagulant ability are pivotal approaches to achieve long-term patency of small-caliber artificial vessels.

**Methods:**

Biocompatible PCL-ECd nanofibers with a core-shell structure were prepared using coaxial electrospinning. PCL served as the shell layer providing mechanical support, while 30% ECd formed the core layer, accelerating endothelialization. Additionally, incorporating 10% heparin into the core layer endows the P-E/H nanofibers with the desired anticoagulant properties. Coaxial-emulsion electrospinning enables sustained release of ECd and heparin from P-E/H. Finally, the in vitro patency of 4 mm diameter P-E/H vascular scaffolds was evaluated using a closed-loop system.

**Results:**

P-E/H nanofibers exhibited enhanced endothelial cell proliferation, superior hemocompatibility, and ideal anticoagulant properties. The in vitro blood flow patency of a 4 mm diameter P-E/H vascular scaffold indicated the absence of any clot or thrombus.

**Conclusion:**

This study proposed a new strategy for developing small-caliber vascular scaffolds with enhanced hemocompatibility and sustained anticoagulant activity.

## 1 Introduction

Cardiovascular diseases represent a significant public health concern, being a leading cause of mortality worldwide (Roth et al., 2020). Allogeneic transplantation and autologous bypass transplantation are pivotal treatments for cardiovascular diseases. However, allogeneic transplantation contains underlying rejection and sources of autologous vessels are limited, which are insufficient to meet substantial clinical demands. Artificial blood vessels as an alternative therapeutic in clinical settings have attracted considerable attention (Li et al., 2023; Liang et al., 2023). Large-caliber (≥6 mm) artificial vessels are extensively employed for cardiovascular reconstruction, vascular bypass procedures, aortic replacement, etc. (Liu et al., 2022). The materials of vascular scaffolds include expanded polytetrafluoroethylene, polyethylene terephthalate, and nylon, which exhibit satisfactory strength and durability under continuous mechanical pressures of blood flow. Although small-caliber (<6 mm) artificial vessels are available for hemodialysis and peripheral arterial disease, thrombus formation and intimal hyperplasia reduce their long-term patency rate (Lawson et al., 2016), which limits their clinical applications (Spadaccio et al., 2016).

The vascular endothelium is primarily composed of endothelial cells, which possess physiological functions including anticoagulation, secretion of growth factors, release of nitric oxide and prevention of endothelial hyperplasia (Cahill and Redmond, 2016). Promoting endothelialization and enhancing anticoagulant effects are key approaches to achieve long-term patency in small-caliber artificial vessels (Li G. et al., 2024; Lu et al., 2021). Recently, a biomimetic small-caliber artificial vessel (2–5 mm) was fabricated containing regenerated cellulose and poly (hydroxyethyl methacrylate), which assembled a dense outer layer and a mesoporous inner layer with a hierarchical structure to mimic native vessels (Tian et al., 2023). Due to the presence of poly (hydroxyethyl methacrylate), a dense hydrophilic surface layer was formed to allow adhesion and growth of endothelial cells. Alternatively, a bilayer vascular scaffold with 1.5 mm lumen diameter was 3D printed to realize endothelialization (Chen et al., 2024). The inner layer was porous with large pore size to facilitate reconstruction of the smooth muscle layer and endothelialization. The surface layer had a lower porosity and minor pore size, providing the desired mechanical strength.

Endothelial cell derivatives (ECd) are secreted by human umbilical vein endothelial cells (HUVECs) and contain vascular endothelial growth factor (VEGF) and bioactive protein substances (Cahill and Redmond, 2016), which can effectively promote endothelialization and provide bioactive support for vascular regeneration and repair. Heparin (Hep) is commonly produced from mast cells and neutrophils in mammals, exhibiting superior anticoagulant and anti-thrombotic properties. It can also promote angiogenesis at an appropriate concentration, providing anticoagulant and pro-regenerative effects for tissue regeneration (Manjunathan et al., 2024; Wang et al., 2024; Liang et al., 2025). Polycaprolactone (PCL) exhibits ideal biocompatibility, making it a promising material for tissue regeneration and repair. Due to the slow degradation rate of PCL, it provides sufficient mechanical support for vascular cell adhesion, growth and tissue regeneration (Wang et al., 2020).

Nanomaterials have a wide range of applications in tissue repair and regeneration (Wa et al., 2025; Chen et al., 2025; Wang et al., 2021; Chaudry et al., 2025; Liu et al., 2024). Their unique physical and chemical properties allow them to simulate the microenvironment of natural tissues. Nanofibers generated through electrospinning technology can simulate the natural extracellular matrix, providing significant advantages in creating artificial vessels. The produced fibrous scaffolds possess a conducive microenvironment for cell attachment, growth and tissue regeneration (Liu et al., 2023). The combination of coaxial electrospinning and emulsion electrospinning imparts multifunctionality to fibers with a core-shell structure compared to conventional electrospun fibers. The emulsion enables the integration of immiscible aqueous and oil phases without compromising the structural integrity and biofunctional properties of the different components (Huang et al., 2025; Inan-Çinkir et al., 2024). Then, the emulsion as a core layer significantly improves loading efficiency and realizes a sustained-release profile of loaded substances. The shell layer maintains ideal mechanical strength and synergistically controls release behavior (Xue et al., 2019). The cooperation of two electrospinning shows potential in sustained-release systems and vascular scaffolds (Wang et al., 2025; Li P. et al., 2024).

To leverage these benefits, PCL-ECd nanofibers with a core-shell structure were prepared through coaxial electrospinning, where PCL, as the shell layer, provided superior mechanical properties, and ECd was incorporated in the core layer to enhance the endothelialization ability for PCL-ECd. Though PCL-ECd suggested comparable biocompatibility and hemocompatibility, it displayed poor anticoagulant properties. Consequently, Hep was incorporated to fabricate P-E/H nanofibers through a combination of coaxial and emulsion electrospinning, with the emulsion serving as the core layer. The anticoagulant and antithrombotic effects of P-E/H were improved with satisfactory cell viability and hemocompatibility. The introduction of emulsion electrospinning endowed a sustained-release profile for P-E/H up to 60 days, which promoted endothelialization for vessel regeneration. The optimized P-E/H was further processed into a tubular scaffold, and its anticoagulant efficacy was determined using an *in vitro* closed-loop system. Overall, the preparation of P-E/H vascular scaffold provided an innovative strategy for fabricating small-caliber artificial vessels.

## 2 Materials and methods

### 2.1 Materials and reagents

Polycaprolactone (PCL, Mn = 80,000) and Span 80 were purchased from Sigma-Aldrich (United States). Hexafluoroisopropanol (HFIP) was purchased from Shanghai Darui Fine Chemicals Co., Ltd. (Shanghai, China). Heparin (Hep), dichloromethane (DCM), and toluidine blue were purchased from Macklin (Shanghai, China). Anticoagulated sheep blood, Calcein AM/PI detection working solution, 4,6-diamidino-2-phenylindole (DAPI), phosphate-buffered saline (PBS), and 4% paraformaldehyde were purchased from Solarbio (Beijing, China). Phalloidin-iFluor 488 (FITC 488) was purchased from Abcam (United Kingdom). Penicillin/streptomycin solution and 0.25% trypsin were obtained from Biosharp (Beijing, China). The Cell Counting Kit-8 (CCK-8) was purchased from Glpbio (United States). Fetal bovine serum (FBS) was purchased from PAN Biotech (Germany). Dulbecco’s Modified Eagle Medium (DMEM) was purchased from Procell (Wuhan, China).

### 2.2 Preparation and characterization of PCL-ECd nanofibers

#### 2.2.1 Preparation of PCL-ECd nanofibers

The preparation of ECd was demonstrated in the Supplementary Material (SI). The prepared ECd was dissolved in double-distilled water at concentrations of 0%, 10%, 20%, 30% and 40% (m/v), separately, with the addition of 5% (m/v) Gel. PCL was dissolved in HFIP to reach a concentration of 10% (m/v). The ECd aqueous solution was used as the core layer, and the PCL solution was used as the shell layer to fabricate PCL-ECd nanofibers by coaxial electrospinning. The production of nanofibers was achieved using a 15 kV positive voltage, a 3 kV negative voltage, a core layer flow rate of 0.05 mL/h, a shell layer flow rate of 2 mL/h, a receiving distance of 18 cm, an 18G/23G coaxial needle, and a flat plate receiver. The nanofibers with different concentrations of ECd were electrospun for 3 h, then placed in a drying oven to remove residual solvent. The nanofibers obtained with different concentrations of ECd were named P-0% E, P-10% E, P-20% E, P-30% E, and P-40% E, respectively.

#### 2.2.2 Morphology of PCL-ECd nanofibers

The morphology was observed using a scanning electron microscope (Regulus 8100, Hitachi Scientific Instruments Co., Ltd., Beijing, China), followed by analysis of the nanofiber diameters using Nano Measurer software. The core-shell structure of PCL-ECd nanofibers was confirmed by a fluorescence microscope. Briefly, FITC 488 was used as a fluorescent agent to label ECd, and then a fluorescence microscope was used to observe the core layer of the nanofibers with an excitation wavelength of 488 nm and an emission wavelength of 520 nm. The images of the core and shell layers for the PCL-ECd nanofibers were merged using Adobe Photoshop 2023 software.

#### 2.2.3 Hydrophilicity of PCL-ECd nanofibers

The hydrophilicity of the PCL-ECd nanofibers was measured by contact angle measurement. The nanofibers were cut into pieces sized 10 mm × 50 mm × 1 mm, followed by fixing on a glass slide to measure the contact angle with deionized water at a drop rate of 5 μL/s.

#### 2.2.4 Tensile properties of PCL-ECd nanofibers

The tensile properties of PCL-ECd nanofibers were evaluated by a universal testing machine (WDW-5G, Jinan Hengshishengda Co., Ltd., Jinan, Shandong, China). The membranes were cut into rectangular pieces sized 5 cm × 1 cm, and the thickness was measured using a microcaliper. The prepared samples were mounted on the clamp of the universal testing machine to test their strength at a rate of 20 mm/min.

#### 2.2.5 Loading efficiency of PCL-ECd nanofibers

The P-30% E was dissolved in 4 mL mixture of DCM and PBS (volume ratio 1:1). The mixture solution was centrifuged at 4,000 rpm for 5 min, followed by removing the supernatant and appropriately diluting. The BCA assay kit was used to determine the concentration of ECd in each nanofiber membrane using a microplate reader at an absorbance of 562 nm. The standard curve was set up, and the loading efficiency of ECd in PCL-ECd nanofibers was calculated according to Equation 1:

$$\text{Load efficiency }\left(\%\right)=\frac{M_{1}}{M_{0}}\times 100\%$$ (1)

Where M_1_ represents the actual weight of ECd, and M_0_ represents the theoretical weight of ECd.

#### 2.2.6 Release profile of PCL-ECd nanofibers

50 mg P-30% E was immersed in a centrifuge tube containing 5 mL PBS and incubated in a thermostatic shaker at 37 °C with 120 rpm. At specific time points, 1 mL PBS was aspirated from the tube and replenished with 1 mL fresh PBS. The 1 mL PBS solution was detected using the BCA assay kit according to the aforementioned method. The release profile was plotted by calculating the cumulative release amount of ECd at different time points.

### 2.3 Cell behaviors of PCL-ECd nanofibers

Cell proliferation of HUVECs seeded on P-0% E, P-10% E, P-20% E, P-30% E, and P-40% E was assessed by CCK-8 assay. Briefly, the obtained nanofiber membranes were adhered to glass slides and placed in a 24-well plate. The plate was sterilized with 75% ethanol vapor for 4 h, followed by exposure to UV irradiation for 30 min. The blank glass slides were set as a control. HUVECs were seeded at 7 × 10^3^ cells/well in 24-well plates and cultured in 400 µL of DMEM supplemented with 10% FBS for 1, 3, and 5 days. Then, the medium was removed and replaced with a medium containing 10% CCK-8 (v/v), and the plate was incubated in an incubator for 2 h. Afterward, 100 µL of medium was transferred to a 96-well plate for measuring the absorbance at 450 nm using a microplate reader (Thermo Scientific Varioskan™ LUX).

The morphology of HUVECs on P-0% E, P-10% E, P-20% E, P-30% E, and P-40% E was observed by an inverted fluorescence microscope on days 3 and 5. Each well was washed three times with PBS, followed by the application of 4% tissue cell fixative to fix HUVECs at 4 °C overnight. Consequently, the fixative was washed with PBS, and 0.1% Triton was added to incubate for 5 min. This was followed by PBS washing and the addition of 1% BSA for 1 h. The F-actin staining solution was diluted with BSA at a 1:2000 volume ratio. This solution was added for 30 min and then washed with PBS. Subsequently, DAPI was added for 5 min at room temperature. The morphology of stained HUVECs was observed and photographed using an inverted fluorescence microscope.

### 2.4 Blood compatibility of PCL-ECd nanofibers

#### 2.4.1 Hemolysis test

Anticoagulated whole blood was centrifuged to collect red blood cells at 2000 rpm for 5 min. The red blood cells obtained were diluted with PBS to create a 4% red blood cell suspension. The mixture of 1 mL PBS, 1 mL 4% red blood cell suspension, and 20 mg produced nanofibers (P-0% E, P-10% E, P-20% E, P-30% E, or P-40% E) was incubated at 37 °C for 3 h. Then, 1 mL of the mixture was transferred to a 1.5 mL centrifuge tube and centrifuged at 2000 rpm for 15 min. A 100 µL supernatant of the mixture was transferred to a 96-well plate to measure the optical density at 545 nm. The mixture of 1 mL of double-distilled water and 1 mL of 4% red blood cell suspension was set up as the positive control. The mixture of 1 mL PBS and 1 mL 4% red blood cell suspension was set up as the negative control. The hemolysis rate was calculated according to Equation 2:

$$\text{Hemolysis rate }\left(\%\right)=\frac{A_{t}-A_{nc}}{A_{pc}-A_{nc}}\times 100\%$$ (2)

Where A_nc_ represents the negative control, and A_pc_ represents the positive control.

#### 2.4.2 Plasma recalcification curve

P-0% E, P-10% E, P-20% E, P-30% E, and P-40% E were attached to glass slides and placed in a 24-well plate, which was then washed three times with PBS. Sheep blood was centrifuged at 2000 rpm for 15 min to obtain platelet-rich plasma (PRP). A total of 500 µL of PRP was added to each well and incubated with the nanofibers for 1 h at 37 °C, while being shaken at 120 rpm. After the incubation period, 100 µL of PRP from each well was transferred to a 96-well plate, followed by the addition of 100 µL of 0.025 M CaCl_2_ solution to each well. The absorbance was measured every 2 min over a 260-min period at a wavelength of 405 nm. Pure PRP served as the negative control, while the mixture of PRP and CaCl_2_ was designated as the positive control.

### 2.5 Preparation and characterization of P-E/H nanofibers

#### 2.5.1 Preparation of P-E/H nanofibers

P-E/H nanofibers were prepared using a water-in-oil emulsion as the core layer through coaxial electrospinning methods. The aqueous phase of the emulsion was prepared by dissolving the optimized ECd concentration at 30% (m/v) and 10% Hep in double-distilled water. The oil phase of the emulsion was prepared by dissolving PCL in DCM at a concentration of 10% (m/v), followed by the addition of Span 80 to the PCL-DCM solution. The aqueous phase solution, which was divided into four parts: containing 30% ECd and 10% Hep, 30% ECd alone, 10% Hep alone, and a control. Each part was added to the oil phase solution, respectively. The volume ratio of oil phase and aqueous phase was 20:1, and the volume ratio of aqueous phase to Span 80 was 5:1. The mixture was vigorously stirred overnight at 1,000 rpm using a magnetic stirrer to form a uniform emulsion. The emulsion serves as the core layer, and the PCL/HFIP solution serves as the shell layer, to fabricate P-E/H nanofibers through coaxial electrospinning for 3 h. The production of nanofibers was achieved using a 15 kV positive voltage, a 3 kV negative voltage, a core layer flow rate of 0.5 mL/h, a shell layer flow rate of 1.5 mL/h, a receiving distance of 18 cm, an 18G/23G coaxial needle, and a flat plate receiver. The produced membranes, containing 30% ECd and 10% Hep, 30% ECd alone, 10% Hep alone, and pure PCL, were named P-E/H, P-E, P-H, and PCL, respectively.

#### 2.5.2 Morphology of P-E/H nanofibers

The morphology observation and loading efficiency of ECd for P-E/H nanofibers were investigated using the aforementioned methods.

#### 2.5.3 Tensile properties of P-E/H nanofibers

The tensile properties of P-E/H nanofibers were evaluated using the same method described in Section 2.2.4, with a strength rate of 20 mm/min.

#### 2.5.4 Loading efficiency of P-E/H nanofibers

P-E/H nanofiber was dissolved in 6 mL mixture of DCM and PBS (volume ratio 1:1), separately. The mixture solution was centrifuged at 4,000 rpm for 5 min, followed by the removal of the supernatant and subsequent dilution. Toluidine blue was used to determine the concentration of Hep in P-E/H and P-H by a microplate reader at 630 nm. The standard curve was set up, and the loading efficiency of Hep in P-E/H nanofibers was calculated according to Equation 3:

$$\text{Load efficiency }\left(\%\right)=\frac{M_{1^{‘}}}{M_{0^{‘}}}\times 100\%$$ (3)

Where M_1’_ represents the actual weight of Hep, and M_0’_ represents the theoretical weight of Hep.

#### 2.5.5 Release profile of P-E/H nanofibers

A 50 mg P-E/H nanofiber was immersed in a centrifuge tube containing 5 mL PBS and incubated in a thermostatic shaker at 37 °C with 120 rpm. At specific time points, 1 mL PBS was aspirated from the tube and replenished with 1 mL fresh PBS buffer solution. The 1 mL PBS solution was detected via toluidine blue by the aforementioned method. The release profiles of ECd and Hep in P-E/H nanofibers were plotted by calculating the cumulative release amounts of ECd and Hep in P-E/H at different time points.

### 2.6 Cell behaviors for P-E/H nanofibers

The cell proliferation and morphology of HUVECs for P-E/H nanofibers were assessed using the aforementioned methods.

The cytotoxicity of P-E/H on HUVECs was assessed through live-dead staining. Briefly, P-E/H was adhered to glass slides and placed in a 24-well plate. The plate was sterilized with 75% ethanol vapor for 4 h, followed by exposure to UV irradiation for 30 min. The blank glass slides were set as a control. HUVECs were seeded at 2 × 10^4^ cells/well in 24-well plates and cultured in 400 µL of DMEM supplemented with 10% FBS for 24 h. Then the medium was removed and washed with PBS, followed by the addition of 200 µL Calcein AM/PI detection working solution to incubate in the dark at 37 °C for 30 min. Observations of live and dead HUVECs were performed under a fluorescence microscope, and the ratio of live to dead cells was determined using ImageJ software.

### 2.7 Blood compatibility of P-E/H nanofibers

#### 2.7.1 Hemolysis test

The hemolysis test of P-E/H nanofibers was conducted using the methods described in Section 2.4.1.

#### 2.7.2 Dynamic coagulation experiment

A dynamic coagulation experiment was conducted to evaluate the anticoagulant effect of P-E/H nanofibers. The P-E/H nanofibers were adhered to glass slides and placed in a 24-well plate to wash three times with PBS. 100 μL anticoagulated whole blood containing 0.025 M CaCl_2_ (Ca^2+^: anticoagulated whole blood = 1:10) was added to each well to incubate at 37 °C for 5, 15, 30, 60, 90, 120 and 180 min, respectively. Then, 2 mL of double-distilled water was added, and the mixture was shaken in a 37 °C incubator for 10 min. The 100 µL resultant supernatant containing ruptured red blood cells was transferred to a 96-well plate, and the absorbance was measured at 540 nm.

#### 2.7.3 Plasma recalcification curve

The anticoagulant effect of P-E/H was also assessed by plotting the plasma recalcification curve. The nanofibers were adhered to glass slides and then placed in a 24-well plate to undergo three washes with PBS. Then, experiments were conducted by adopting the methods outlined in Section 2.4.2. The absorbance was measured every 2 min throughout 320 min at 405 nm. Pure PRP served as the negative control, and PRP with CaCl_2_ served as the positive control.

### 2.8 Preparation of P-E/H vascular scaffolds and evaluation of blood flow patency

The preparation of P-E/H vascular scaffolds was conducted by modifying the methods in Section 2.5.1. Specifically, the flat plate receiver was replaced with a stainless steel metal rod receiver with diameters of 1 mm, 2 mm, 3 mm, 4 mm, 5 mm, and 6 mm. The rolling speed of the receiver was set at 600 rpm. The morphology of the cross-section and longitudinal section of a 4 mm diameter P-E/H vascular scaffold was observed using SEM.

The anticoagulant efficacy of tubular scaffolds was determined by an *in vitro* blood circulation test. A closed-loop system, comprising a peristaltic pump, centrifuge tubes, silicone tubing, and P-E/H vascular scaffolds, was utilized to investigate the anticoagulant properties of the P-E/H vascular scaffold by simulating the *in vitro* blood circulation process. A peristaltic pump was used to simulate venous blood flow, with a flow rate of 20 mL/min. The P-E/H vascular scaffolds were removed after 3 and 5 days of blood circulation, respectively. The residual blood from the inner surface of P-E/H vascular scaffolds was washed with PBS, and the cross-section and longitudinal sections of the scaffolds were photographed.

### 2.9 Statistical analysis

The multiple comparison procedures between groups were performed using one-way ANOVA with Origin, and each group was repeated at least three times. Statistical results were expressed as means ± standard deviation (SD). To observe the significance of differences between the test groups. Student’s t-test was used for all two-by-two comparisons. A value of ^*^ *P* < 0.05 represents the lowest significance level, ^**^ *P* < 0.01 represents a moderate significance level, and ^***^ *P* < 0.001 represents the highest significance level.

## 3 Results and discussion

### 3.1 Characterization of PCL-ECd nanofibers

Endothelial cells secrete various functional proteins, such as fibronectin and collagen, which constitute the primary regulatory network of the angiogenic microenvironment, along with VEGF. As a proangiogenic factor, VEGF modulates endothelialization by regulating the proliferation and migration of endothelial cells through activation of the PI3K/AKT signaling pathway (Krüger-Genge et al., 2019). The extracted ECd as crude conditioned medium lysate of HUVECs contains 0.35 ± 0.019 mg endothelial cell-secreted proteins and 41.96 ± 20.39 pg VEGF in ECd to promote endothelial proliferation (Supplementary Figure S1). In this study, coaxial electrospinning technology was employed to fabricate PCL-ECd nanofibers with a core-shell structure. As demonstrated in SI, the core layer and shell layer flow rates were set to 0.1–1 mL/h, 0.1–2 mL/h, and 0.05–2 mL/h, named as PE1, PE2, and PE3, respectively. The morphology of resultant nanofibers was displayed in Supplementary Figure S2, suggesting the spinnability of PE1, PE2, and PE3. Although the obtained fibers exhibited a random distribution, PE2 and PE3 showed more uniform thickness, smoother surfaces, and a more satisfying morphology compared to PE1. The loading efficiency of ECd in PE1, PE2 and PE3 was measured as 50.68% ± 8.05%, 74.44% ± 12.68%, and 81.68% ± 10.75%, respectively (Supplementary Figure S3). The superior loading efficiency of PE2 and PE3 may have contributed to the faster flow rate of the shell layer, allowing it to stretch and trap nanofibers, thereby facilitating the encapsulation of the core layer. The highest loading efficiency of PE3 may be relative to the lower flow rate of the core layer compared to PE2, which allows the core layer solution to fill into the interior fiber with the fast flow rate of the shell layer, resulting in a reduction of uneven distribution of nanofibers for the core layer and improvement of loading efficiency for the core layer.

**FIGURE 1:**
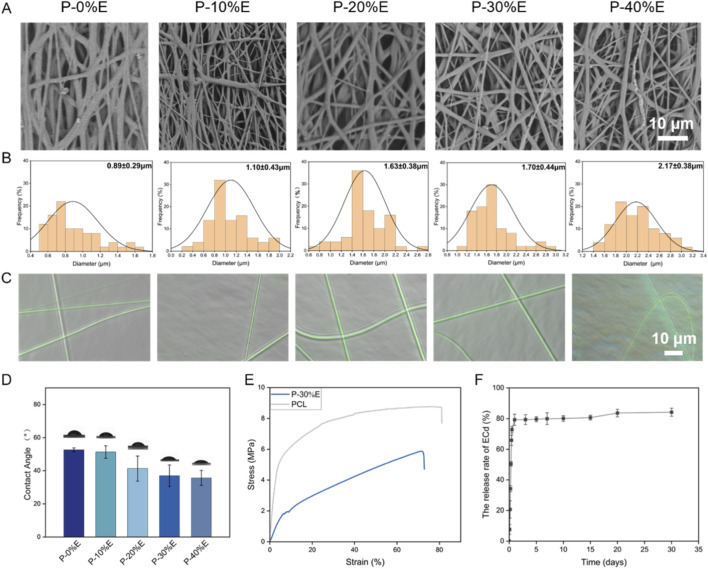
**(A)** SEM image of PCL-ECd nanofibers; **(B)** Fiber diameter distribution image and **(C)** Fluorescence image of PCL-ECd nanofibers featuring a core-shell structure, which contains varying concentrations of ECd (the core layer of the nanofiber membrane showed green fluorescence, and the shell layer was colorless); **(D)** The water contact angles of PCL-ECd nanofibers containing varying concentrations of ECd; **(E)** The stress-strain curves of PCL-ECd nanofibers containing 30% ECd and pure PCL nanofiber; **(F)** Sustained-release curves of P-30% E.

Subsequently, PCL-ECd nanofibers with a core-shell structure were prepared following the parameters of PE3. The morphology and fiber diameter distribution of PCL-ECd nanofibers are shown in Figure 1A. The produced random nanofibers with different amounts of ECd featured smooth surfaces. Figure 1B indicated that the average diameter of P-0% E, P-10% E, P-20% E, P-30% E, and P-40% E was 0.89 ± 0.29 µm, 1.10 ± 0.43 µm, 1.63 ± 0.38 µm, 1.70 ± 0.44 µm, and 2.17 ± 0.38 µm, respectively. The increase in ECd concentration is correlated with the diameter of the fiber. FITC could emit green fluorescence at specific wavelengths by binding with functional groups of proteins (Restan et al., 2019). FITC was selected to confirm the core-shell structure for PCL-ECd nanofibers. As shown in Figure 1C, the core layer displayed green fluorescence as FITC binding proteins within ECd, indicating the successful preparation of a core-shell structure for PCL-ECd nanofibers. The addition of ECd improved the hydrophilicity of fibrous membranes compared to the pure PCL nanofiber membrane (Figure 1D). It could be explained by the formation of hydrogen bonds between the hydroxy or carboxyl group within ECd and the hydroxy group within water and the van der Waals force. The parameters for producing P-30% E were selected to conduct the following experiments, which were described in detail below. The tensile properties were evaluated through stress-strain curves, which compared the tensile strength of P-30% E and pure PCL nanofibers (Figure 1E). The tensile strength of P-30% E was 5.44 ± 0.37 MPa, which was lower than PCL nanofibers at 8.21 ± 0.48 MPa. Though the presence of ECd weakened the mechanical properties of the prepared fibrous membrane, the tensile strength of P-30% E is over the tensile modulus for human coronary arteries (0.7–2.1 MPa) (Norouzi and Shamloo, 2019). It could meet the mechanical performance requirements for small-caliber artificial vessels. The loading efficiency of P-30% E was measured as 75.14% ± 1.58%. P-30% E nanofibers exhibited a fast release in 24 h at 79.29% ± 3.63% and cumulative release up to 84.22% ± 2.62% after 30 days (Figure 1F). The release profile of P-30% E failed to meet the long-term sustained-release requirements. Then, coaxial-emulsion electrospinning was employed to prepare nanofibers for subsequent experiments, aiming to achieve sustained-release behavior.

### 3.2 *In vitro* assessment for PCL-ECd nanofibers

**FIGURE 2:**
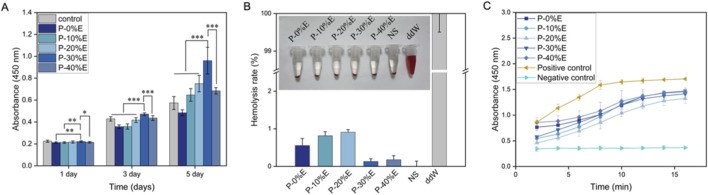
**(A)** Cell proliferation of HUVECs co-cultured with P-0% E, P-10% E, P-20% E, P-30% E or P-40% E; **(B)** Hemolysis rate and **(C)** Plasma recalcification curves of P-0% E, P-10% E, P-20% E, P-30% E or P-40% E (negative control: saline; positive control: double-distilled water). *P < 0.05, **P < 0.01 and ***P < 0.001 indicate that the differences between the compared groups are statistically significant.

The biocompatibility of PCL-ECd nanofibers with different amounts of ECd was assessed using HUVECs. P-0% E, P-10% E, P-20% E, P-30% E and P-40% E indicated good biocompatibility over 5 days, where P-30% E and P-40% E showed significant difference with P-0% E, P-10% E and P-20% E (Figure 2A). P-30% E exhibited a faster proliferation behavior than P-40% E, suggesting that an appropriate ECd concentration is favorable for HUVECs proliferation.

Artificial vessels, as medical devices that come into contact with blood, require superior hemocompatibility. Otherwise, they may lead to severe damage to red blood cells, hemolysis, or even the formation of thrombus (Xiang et al., 2024). The hemolysis test is used to evaluate damage to red blood cells caused by material, and the hemolysis rate demonstrates damage severity (Lu et al., 2021). In this test, double-distilled water served as the positive control, and saline solution served as the negative control. As shown in Figure 2C, the supernatant of P-0% E, P-10% E, P-20% E, P-30% E, P-40% E, and the negative control was colorless and transparent with red blood cells precipitated at the bottom. In comparison, sedimentation was not observed after centrifugation, as red blood cells were ruptured in the positive control. The quantitative analysis indicated the hemolysis rate in material groups was below 2%, indicating mild hemolysis. GB/T 16,886.4–2022 requires the hemolysis rate of medical devices to be below 5%, which suggests the promising potential of PCL-ECd nanofibers in clinical settings (Ge et al., 2019). The plasma recalcification time was determined to assess anticoagulant properties of P-0% E, P-10% E, P-20% E, P-30% E, and P-40% E. Calcium ions were added into plasma to induce coagulation, and the coagulation rate was measured by observing the half-coagulation time and the slope of the recalcification curve (Ma et al., 2024; Xu et al., 2023a). Comparing with the negative control, the absorbance of other groups was increased, suggesting the appearance of coagulation. However, material groups failed to prolong coagulation time, demonstrating poor anticoagulant efficacy of PCL-ECd nanofibers.

### 3.3 Characterization of P-E/H nanofibers

To improve the anticoagulant and anti-thrombotic properties of PCL-ECd nanofibers, Hep was involved in fabricating P-E/H nanofibers with a core-shell structure. The morphology of P-E/H, P-E, P-H and pure PCL was displayed in Figure 4A, illustrating a smooth surface and uniform diameter for P-E/H. As shown in Figure 4B, the average diameters of P-E/H, P-E, P-H, and PCL were 631.3 ± 198.4 nm, 550.6 ± 156.57 nm, 459.7 ± 106.2 nm, and 1.074 ± 0.435 µm, respectively. Although nanofibers introduced two different components, the average diameters of P-E/H were smaller than those of pure PCL. The introduction of Hep and oil-in-water emulsion narrowed the diameter of PCL-ECd nanofibers. The addition of Span 80 enhanced the interface compatibility between the aqueous phase and the oil phase in the emulsion, resulting in a more uniform bonding of the interface between the core layer and the shell layer during the electrospinning process and stretched jet under the electric field. Furthermore, ECd and Hep were encapsulated within the aqueous phase by PCL in the oil phase to form dispersed droplets, rather than a continuous aqueous phase solution, which significantly reduced the viscosity of the core layer, allowing for the stretching of nanofibers with smaller diameters.

**FIGURE 3:**
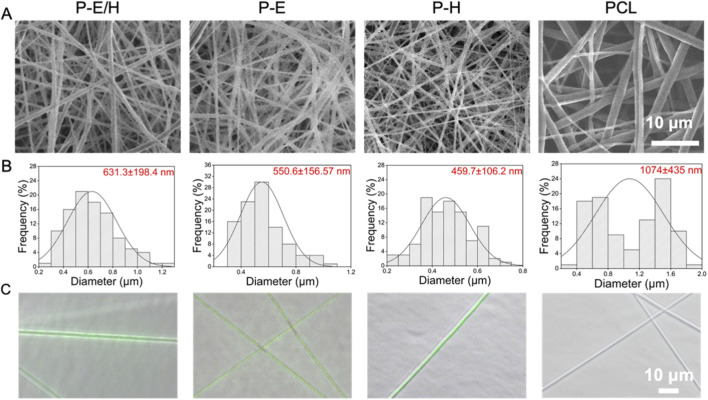
**(A)** SEM image of P-E/H, P-E, P-H, and PCL nanofibers; **(B)** Fiber diameter distribution image and **(C)** Fluorescence images of P-E/H, P-E, P-H, and PCL (The core layer of nanofiber membrane was green fluorescence, and the shell layer was colorless).

**FIGURE 4:**
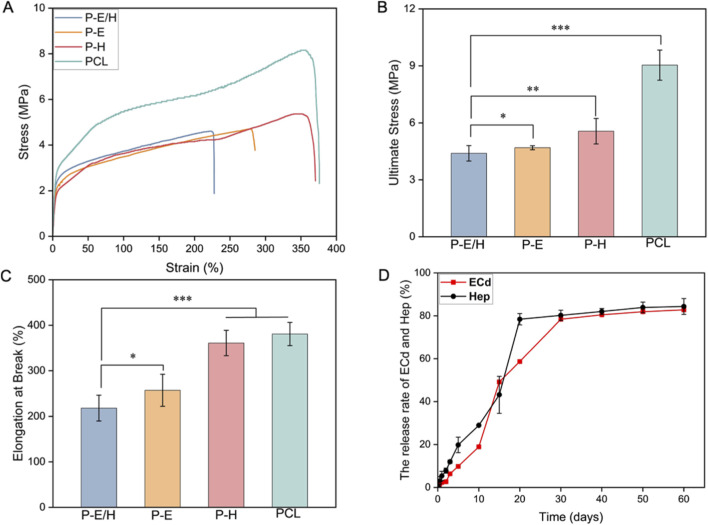
**(A)** Stress-strain curve, **(B)** Ultimate stress and **(C)** Elongation at break of P-E/H; **(D)** Sustained-release profile of ECd and Hep in P-E/H nanofiber. *P < 0.05, **P < 0.01, ***P < 0.001 indicate that the differences between the compared groups are statistically significant.

The successful preparation of a core-shell structure for P-E/H nanofibers was confirmed following the aforementioned approach. Briefly, FITC mixed with ECd and Hep in the core layer showed green fluorescence, while the shell layer was uncolored (Figure 3C). The tensile properties of P-E/H, P-E, P-H, and PCL were examined through stress-strain curves (Figure 4A). Due to the reduction of PCL within the core layer, the tensile strength of P-E/H, P-E, and P-H was lower than that of PCL at 9.04 ± 0.79 MPa. And the ultimate stress of P-E/H, P-E, and P-H reached 4.39 ± 0.41 MPa, 4.69 ± 0.11 MPa, and 5.56 ± 0.67 MPa, respectively, which was weaker than PCL at 9.04 ± 0.79 MPa (Figure 4B). The elongation at break of P-E/H and P-E was 218.10% ± 28.27% and 257.14% ± 35.18%, respectively (Figure 4C). It was lower than P-H (361.12% ± 27.96%) and PCL (380.94% ± 25.32%). Though the tensile strength of P-E/H lowered to 4.6 ± 0.2 MPa, it is still greater than that strength desired for human coronary arteries at 0.7–2.1 MPa. Herein, P-E/H exhibited ideal mechanical performance to meet the requirements for small-caliber artificial vessels. The loading efficiency of ECd and Hep in P-E/H was assessed as 78.51% ± 5.77% and 65.14% ± 1.58%, respectively. The combination of coaxial-emulsion electrospinning prolonged the release time of ECd and Hep, achieving a sustained release of up to 60 days (Figure 4D). The cumulative release of ECd in P-E/H reached 78.41% ± 0.03% on day 30, and Hep in P-E/H was cumulated to 78.46% ± 2.42% for 20 days, followed by a slow-release behavior. It may be assigned with a water-in-oil emulsion, where the aqueous phase is dispersed as droplets in the oil phase to hinder the release of ECd and Hep from the aqueous phase. Moreover, the core-shell structure of nanofibers exerted a synergetic effect in impeding the release of ECd and Hep, as they were encapsulated in the core layer. Additionally, the shell layer of nanofibers may contain tiny pores, allowing the oil phase of the emulsion to fill them and form a denser shell layer, thereby reducing the leakage of ECd and Hep.

### 3.4 *In vitro* assessment for P-E/H nanofibers

**FIGURE 5:**
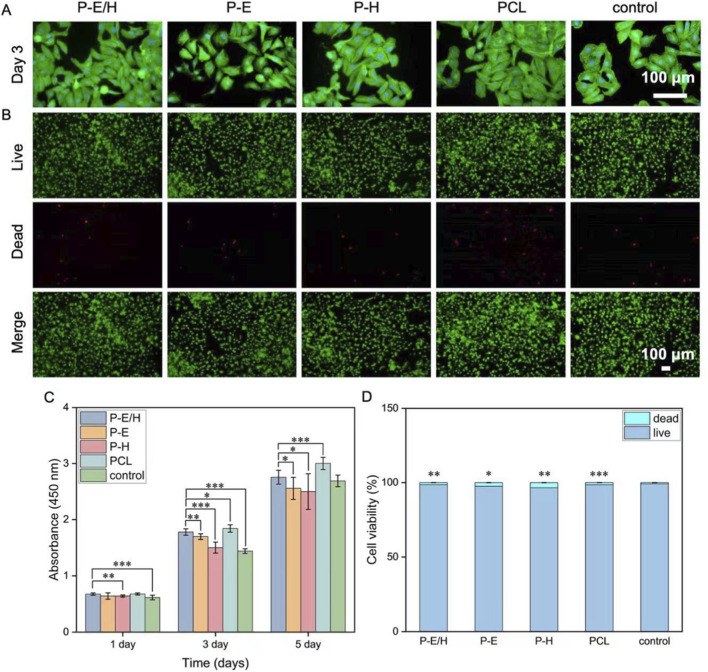
**(A)** Fluorescence images of HUVECs co-cultured with P-E/H for 3 days (Blue: DAPI for staining cell nucleus; Green: Phalloidin-iFluor 488 for staining cytoskeleton); **(B)** Live/dead staining images of HUVECs co-cultured with P-E/H for 24 h (Green: live; Red: dead); **(C)** Cell proliferation of HUVECs co-cultured with P-E/H; **(D)** Quantitative analysis of the number of viable and dead cells of HUVECs co-cultured with P-E/H for 24 h *P < 0.05, **P < 0.01, ***P < 0.001 indicate that the differences between the compared groups are statistically significant.

The biocompatibility of P-E/H, P-E, P-H, and PCL was assessed using HUVECs. Figure 5A shows the staining results of HUVECs co-cultured on different fibers for 3 days, where cell nuclei were stained blue, and the cytoskeleton was stained green. HUVECs cultured on P-E/H, P-E, P-H, and PCL exhibited a pebble-like morphology, overlapping with that of the control. Cell compatibility was further evaluated using live/dead cell staining. Live cells exhibited green fluorescence, while dead cells exhibited red fluorescence (Figure 5B). The fluorescence images of HUVECs co-cultured with P-E/H, P-E, P-H, and PCL for 24 h demonstrated that P-E/H possessed good cell compatibility. The number of live cells for produced nanofibers exceeded 96%, demonstrating no adverse effects on cell viability for P-E/H, P-E, P-H, and PCL (Figure 5D). The absorbance of HUVECs co-cultured with P-E/H, P-E, P-H and PCL was measured for 1, 3, and 5 days (Figure 5C). There was a cell proliferation of HUVECs co-cultured with obtained fibrous membranes over 5 days, suggesting a desired cell compatibility for P-E/H, P-E, P-H and PCL. P-E/H showed superior cell proliferation ability compared with PCL on day 3 (*P* = 0.025) and day 5 (*P* = 0.00021). P-E/H showed a pronounced cell proliferation profile compared to P-E (*P* = 0.0025 on day 3 and *P* = 0.0251 on day 5) and P-H (*P* = 0.0017 on day 1, *P* = 0.000003 on day 3 and *P* = 0.023 on day 5), which may contribute to ECd and Hep synergistically exerting promotion effects for HUVECs proliferation (Wang et al., 2015). PCL also showed acceptable cell proliferation that may be attributed to the high sensitivity of HUVECs to their local microenvironment. HUVECs responded to topographical cues of micron-scale PCL, facilitating cell spreading and proliferation (Ahmed et al., 2018).

### 3.5 Blood compatibility of P-E/H nanofibers

**FIGURE 6:**
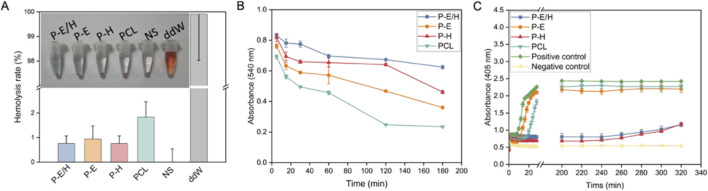
**(A)** Hemolysis rate, **(B)** Dynamic coagulation curve and **(C)** Plasma recalcification curve of P-E/H nanofiber membrane.

The hemolysis test was employed to assess the damage of red blood cells caused by P-E/H, P-E, P-H and PCL. In this test, double-distilled water served as the positive control, and saline solution served as the negative control. As shown in Figure 6A, P-E/H, P-E, P-H, PCL, and the negative control did not exhibit significant changes, as the supernatant was transparent with red blood cells precipitated at the bottom. In contrast, the red blood cells of the positive control were ruptured, with the absence of sedimentation after centrifugation. Based on the quantitative analysis of supernatant, the hemolysis rates in the material groups and the negative control were below 2% (Figure 6A). It demonstrated P-E/H, P-E, P-H and PCL lead to mild hemolysis, being classified as non-hemolytic biomaterials.

The anticoagulant effect of P-E/H, P-E, P-H, and PCL was evaluated through a dynamic coagulation experiment to determine the coagulation of anticoagulated whole blood on the obtained nanofibers. Specifically, the red blood cells that were not involved in the thrombus were lysed in double-distilled water, and the absorbance change was then determined (Li G. et al., 2024; Xu et al., 2023a). Figure 6C shows the absorbance change of P-E/H, P-E, P-H and PCL contacting with whole blood at different time points. At 180 min, the absorbance ranked as P-E/H > P-H > P-E > PCL. The highest absorbance for P-E/H and a slower decline in the coagulation curve, illustrating that the addition of hep improved the anticoagulant effect of P-E/H. The plasma recalcification time was determined to further verify the anticoagulant performance of P-E/H, P-E, P-H and PCL. As shown in Figure 6B, P–E, PCL, and positive control exhibited calcification with the addition of CaCl_2_. In comparison, P-E/H and P-H calcified until 240 min, suggesting the presence of Hep improved the anticoagulant properties of P-E/H and P-H.

### 3.6 Characterization of P-E/H vascular scaffolds

**FIGURE 7:**
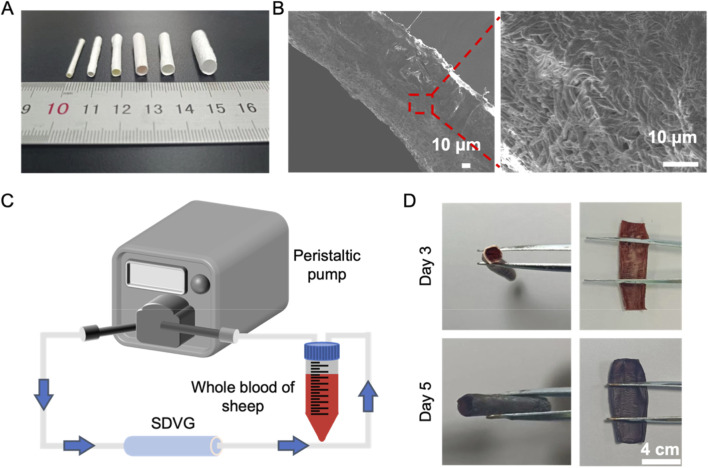
**(A)** Digital images of P-E/H vascular scaffolds with different diameters at 1, 2, 3, 4, 5 and 6 mm; **(B)** Cross-sectional view of P-E/H vascular scaffold; **(C)** Schematic diagram of P-E/H vascular scaffold *in vitro* blood circulation test and **(D)** Digital images of *in vitro* blood circulation test for P-E/H vascular scaffold (diameter with 4 mm) after 3 and 5 days.

As shown in Figure 7A, P-E/H vascular scaffolds with different diameters were successfully fabricated. Figure 7B reveals that longitudinal and transverse sections of P-E/H with 4 mm diameters show a disordered arrangement without bead-like morphology. This structure enhances flexibility for vascular scaffolds and promotes cell proliferation. A closed-loop system was used to evaluate the anticoagulant properties of P-E/H vascular scaffolds by simulating the *in vitro* blood circulation process (Figure 7C). Figure 7D shows images of the 4 mm P-E/H vascular scaffold *in vitro* circulation test on days 3 and 5. Results indicate that after 3 and 5 days of circulation testing, the outer surface of the vascular scaffold remained smooth, and no thrombus was observed on the inner wall of the scaffold. Therefore, the P-E/H vascular scaffold exhibits anticoagulant properties over a certain period, preliminarily mimicking the functionality of natural vessels (Xu et al., 2023b). However, our study lacks *in vivo* patency data to verify the long-term performance of P-E/H vascular scaffolds. Future animal experiments will be conducted to further compare the performance of P-E/H vascular scaffolds with natural vessels.

## 4 Conclusion

In this study, coaxial electrospinning was employed to prepare core-shell structured nanofibers. PCL as a shell layer provided excellent mechanical properties, and ECd as a core layer contained various active substances to promote endothelial cell proliferation and improve cell compatibility. A 30% ECd concentration was considered optimal for promoting cell proliferation, exhibiting spinnability, cell compatibility, and hemocompatibility in PCL-ECd nanofibers. Subsequently, Hep was involved in improving the anticoagulant properties of PCL-ECd nanofibers. Water-in-oli emulsion was combined with coaxial electrospinning to fabricate P-E/H nanofibers with a sustained-release profile up to 60 days. ECd and Hep were loaded into the core layer, where Hep displayed anticoagulant and regenerative vascular effects. The biocompatibility, anticoagulant properties, and mechanical properties of P-E/H were evaluated using live-dead staining, a hemolysis test, a dynamic coagulation experiment, a plasma recalcification curve, and a tensile strength test. The *in vitro* circulation test also proved that the addition of Hep prevents the formation of a clot and thrombus in a 4 mm P-E/H vascular scaffold. The prepared P-E/H vascular scaffolds meet clinical requirements for peripheral arterial diseases and hemodialysis, addressing issues of thrombosis and insufficient endothelialization in small-caliber artificial vessels. However, this study only simulated blood flow environments *in vitro* and has not yet explored factors such as *in vivo* immune rejection, dynamic hemodynamics, and vascular remodeling. To assess the feasibility of clinical translation, future studies should evaluate long-term patency, tissue integration, and safety in large animal models.

## Data Availability

The original contributions presented in the study are included in the article/Supplementary Material, further inquiries can be directed to the corresponding authors.
