# Supplementary material for: Fabrication and characterization of small-caliber nanofibrous vascular scaffolds with sustained release of endothelial cell derivatives and heparin
Source: Front Bioeng Biotechnol. 2026 Feb 26;14:1746603. doi: 10.3389/fbioe.2026.1746603 (PMC12979498; doi:10.3389/fbioe.2026.1746603)
Supplement: Supplementary file 1 [file Supplementaryfile1.docx]

**Supplementary information**

**Fabrication and characterization of small-caliber nanofibrous vascular scaffolds with sustained release of endothelial cell derivatives and heparin**

Ying Wang^1,2,#^, Yawen Wang^3,#^, Qihan Yuan^4^, Jiaoyan Qiu^1^, Jing Wang^1,2^, Yuanfei Wang^2^, Manfei Fu^1,*^, Tong Wu^1,2,*^

^1^ Medical Research Center, The Affiliated Hospital of Qingdao University, Qingdao University, Qingdao 266000, China

^2^ Shandong Key Laboratory of Medical and Health Textile Materials, Collaborative Innovation Center for Eco-textiles of Shandong Province and the Ministry of Education, College of Textile & Clothing, Qingdao University, Qingdao 266071, China

^3^ School of Rehabilitation Sciences and Engineering, University of Health and Rehabilitation Sciences, Qingdao 266113, China

^4^ Qingdao Traditional Chinese Medicine Hospital, Qingdao Hiser Hospital Affiliated of Qingdao University, Qingdao 266000, China

^#^ Ying Wang and Yawen Wang contribute equally to this work.

Corresponding authors:

Manfei Fu: [j201745776@outlook.com](mailto:j201745776@outlook.com)

Tong Wu: [twu@qdu.edu.cn](mailto:twu@qdu.edu.cn)

*Extraction of ECd*

Prepare a complete medium using DMEM high-glucose medium containing 10% FBS. Seed 1 × 10^5^ endothelial cells into a culture dish and add 6 mL of complete medium. Incubate the cells at 37°C in a cell culture incubator containing 5% CO₂ for three days. After this incubation period, aspirate the conditioned medium and collect the supernatant by centrifuging it at 900 rpm for 5 minutes. Freeze the supernatant overnight at -80°C using an ultra-low-temperature freezer. Finally, lyophilize the frozen supernatant into a solid using a freeze dryer to obtain the final product.

*Protein content in ECd*

A 1 mg/mL solution was prepared from the ECd powder using PBS. The absorbance of the protein in this solution was measured using a BCA reagent kit. The absorbance values were then used in conjunction with a BSA standard curve to determine the protein content in the ECd solution. To ensure reproducibility and stability, three different batches were tested. Firstly, prepare the BCA working solution. Calculate the quantities of the standard samples and the test samples. Combine the BCA reagent with the Cu reagent in a 50:1 volume ratio to create the working solution, and mix thoroughly. Then dilute the standard samples using PBS to achieve final concentrations of 1 mg/mL, 0.5 mg/mL, 0.25 mg/mL, 0.125 mg/mL, 0.0625 mg/mL, and 0.03125 mg/mL. Add 50 µL of each standard sample concentration to a 96-well plate, followed by the rapid addition of 200 µL of the working solution. After incubating the sample at 37°C for 30 minutes, the absorbance at 562 nm was measured using an ELISA reader. A BSA standard curve was created, plotting BSA concentration (0 - 1 mg/mL) on the x-axis and absorbance on the y-axis.

*VEGF content in ECd*

Prepare the standard gradient working solution, the biotinylated antibody working solution, the enzyme conjugate working solution, and the 1 × washing solution according to the pre-calculated values. The experimental steps are outlined as follows: Remove the necessary number of strips from the aluminum foil bag that has been equilibrated at room temperature for 10 minutes. Next, add the samples and standard solutions of varying concentrations to the corresponding wells, using a volume of 100 µL per well. For the blank wells, add only 100 µL of universal diluent. Cover the microplate with a sealing membrane and incubate it at 37°C for 60 minutes. After incubation, remove the microplate and aspirate the liquid without washing the wells. Then, add 100 µL of the biotinylated antibody working solution to each well. Again, cover with a sealing membrane and incubate at 37°C for an additional 60 minutes. Remove the liquid from the wells, and add 300 µL of washing solution to each well. One minute later, discard the washing solution and blot dry on absorbent paper. Repeat this washing step three times. Next, add 100 µL of enzyme conjugate working solution to each well, cover with a sealing membrane, and incubate at 37°C for 30 minutes. Remove the liquid and repeat the washing step five times. Add 90 µL of substrate to each well, cover with a sealing membrane, and incubate at 37°C in the dark for 15 minutes. Remove the microplate, add 50 µL of stop solution to each well, and quickly measure the absorbance at 450 nm. Three different batches were tested to ensure experimental reproducibility and stability.

*Preparation of the heparin solution*

Determination of the concentration of heparin solution using the toluidine blue method involves the following steps: First, prepare a 0.001% toluidine blue working solution by weight. Then, dilute the sodium heparin solution to different concentrations using a PBS solution. Add 2 mL of each heparin solution concentration to a 10 mL centrifuge tube, followed by 3 mL of the toluidine blue working solution. Allow the reaction to proceed thoroughly at 37°C for 2 hours. Filter the mixture through a 0.22 µm filter, then measure the absorbance at 630 nm using an ELISA reader. Three samples were tested in each group. Plot a heparin standard curve (0 - 50 µg/mL) with heparin concentration as the x-axis and absorbance as the y-axis.

*Preparation of PCL-ECd nanofibers*

PCL-ECd nanofibers were prepared using a coaxial electrospinning method. First, ECd was dissolved in double-distilled water at a concentration of 10%, and 5% Gel was added to increase the viscosity of the spinning solution and enhance spinnability. PCL was dissolved in HFIP at a concentration of 10% (m/v). ECd solution and gel solution serve as the core solution, while PCL solution functions as the shell solution. The coaxial electrospinning apparatus is used for the process, with spinning parameters set to positive pressure of 15 kV, negative pressure of 3 kV, core layer flow rate and shell layer flow rate set to 0.1 - 1 mL/h, 0.1 - 2 mL/h, 0.05 - 2 mL/h, distance of 18 cm, using a 18G - 23G coaxial needle, and a flat plate receiving device. Each parameter was spun for 3 hours. The prepared nanofibers were placed in a drying oven to remove any residual HFIP that had not evaporated during the process. The nanofibers prepared under different parameters were named PE1, PE2, and PE3, respectively.

*Characterization of PCL-ECd nanofibers*

SEM was used to observe the surface microscopic morphology of nanofibers in each group. First, the nanofibers were cut into 3 mm squares. Then, conductive glue was used to fix them on the sample stage. Finally, after spraying gold for 20 seconds under vacuum conditions using a gold spraying station, the surface microscopic morphology of the fibers was observed using SEM, with an acceleration voltage set to 10 kV.

*Loading rate of ECd*

Nanofibers of the PE1, PE2, and PE3 groups were prepared using the above method, with an electrospinning time of 1 hour. After drying, the nanofibers were completely dissolved in 4 mL of a mixture of dichloromethane and PBS (volume ratio of 1 : 1) and centrifuged at 4000 rpm for 5 minutes. The upper layer of liquid was then removed, appropriately diluted, and measured. The concentration of ECd in the PBS solution was detected using a BCA assay kit. The content of ECd in PBS was obtained via a standard curve. The loading rate was calculated using formula (1):

$load rate\left（ \% \right）=\frac{M1}{M0}\times100\%$ （1）

Among them, M1 and M0 are the actual weight and theoretical weight of ECd in each group of the nanofibers, respectively.


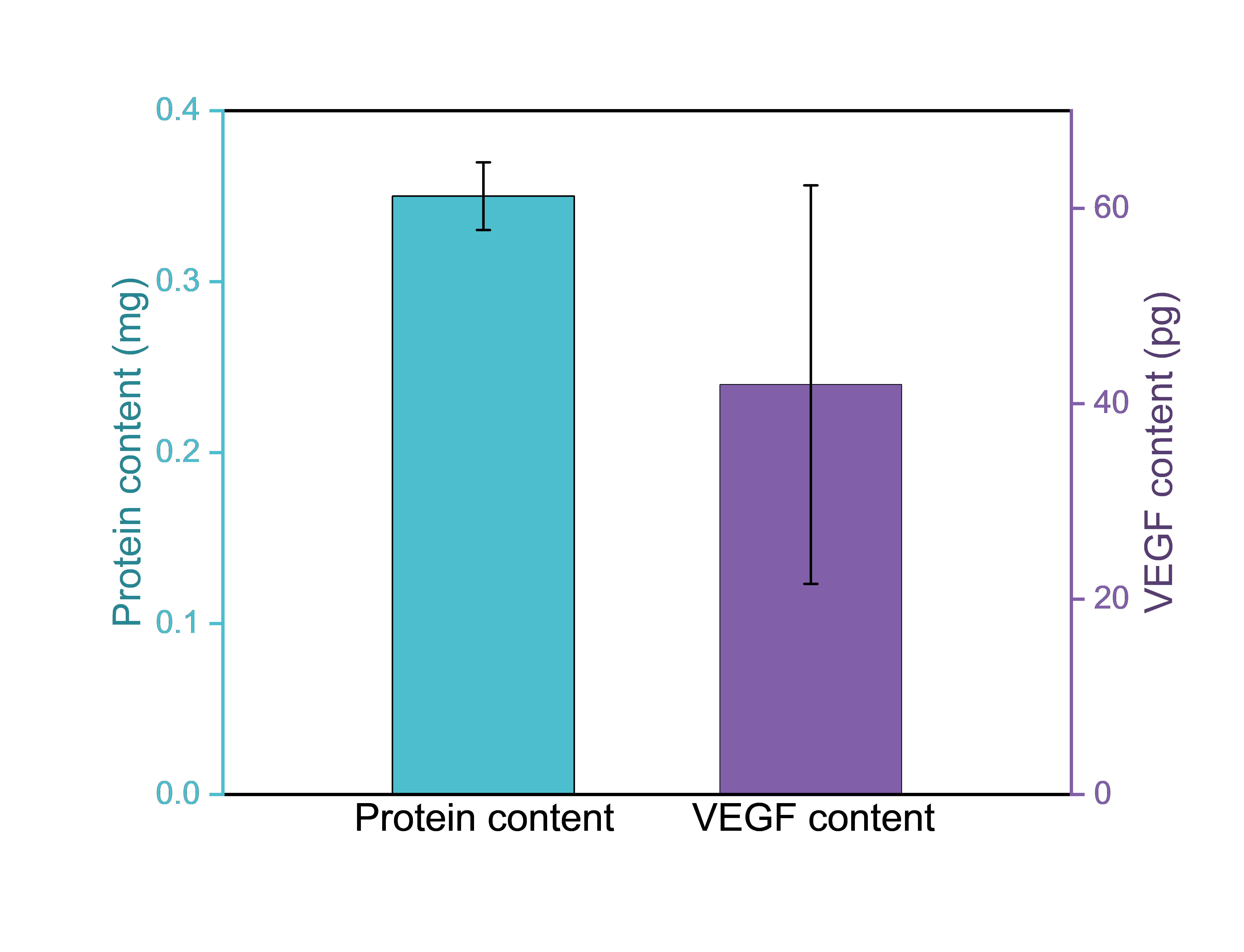


Figure S1. Content of protein and VEGF in ECd


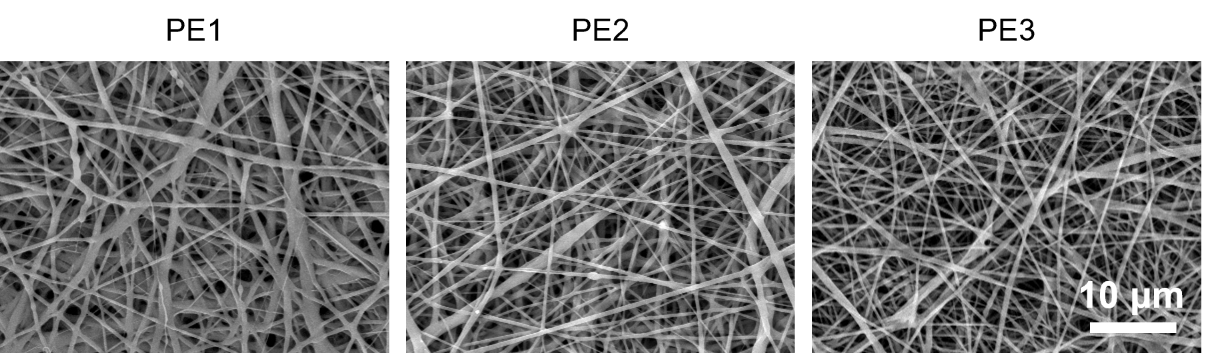


Figure S2. SEM image of PCL-ECd nanofibers


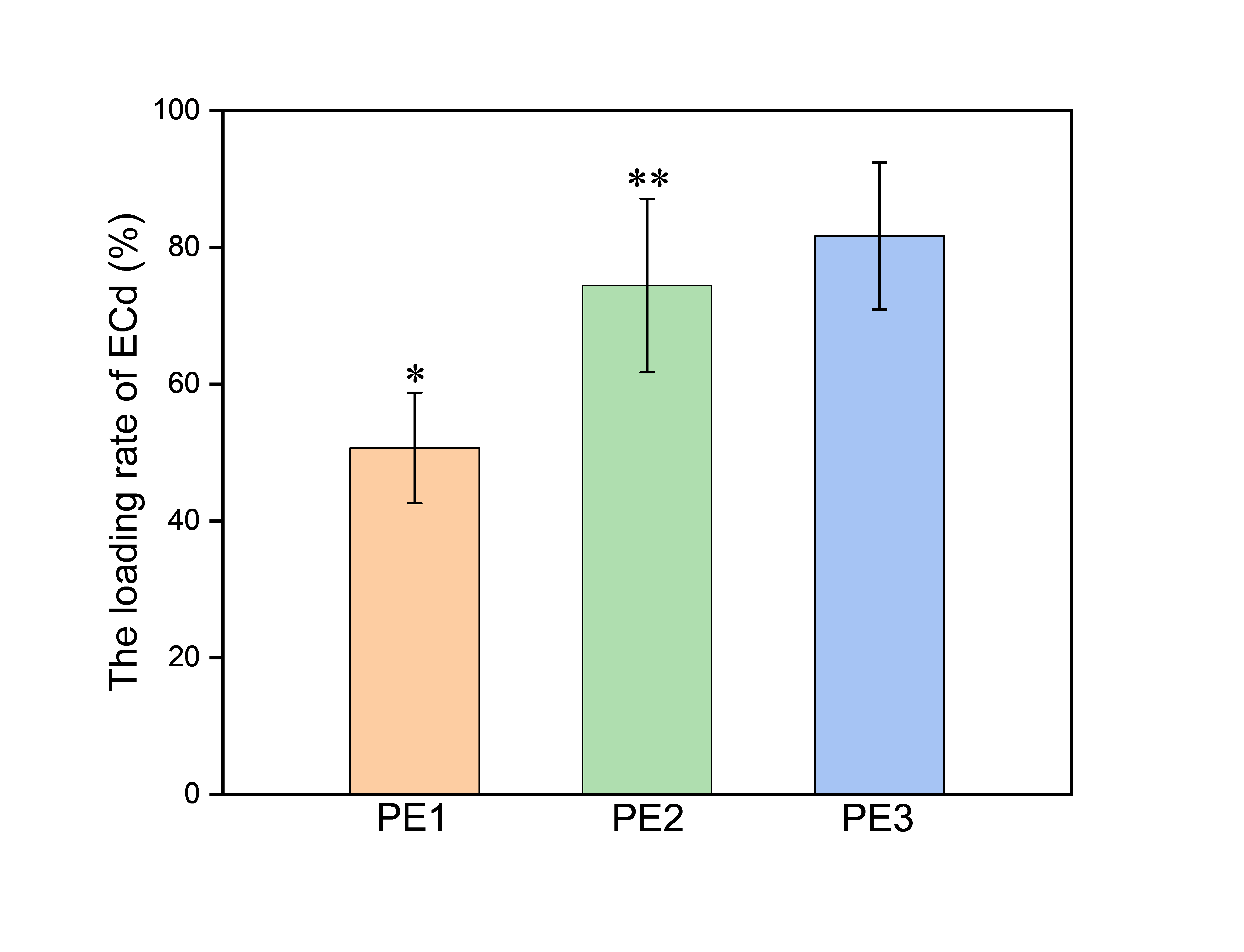


Figure S3. Loading rate of ECd in PCL-ECd nanofibers (Compared with the PE3 group, **P*  <  0.05, ***P*  <  0.01 indicates that the difference between groups is statistically significant).
